# Can extended health communication improve newly settled refugees’ health literacy? A quasi-experimental study from Sweden

**DOI:** 10.1093/heapro/daae015

**Published:** 2024-03-02

**Authors:** Maissa Al-Adhami, Natalie Durbeej, Achraf Daryani, Josefin Wångdahl, Elin C Larsson, Raziye Salari

**Affiliations:** Child Health and Parenting (CHAP), Department of Public Health and Caring Sciences, Uppsala University, Husargatan 3, 752 37 Uppsala, Sweden; Research and Learning for Sustainable Development and Global Health (SWEDESD), Department of Women’s and Children’s Health, Uppsala University, Hammarskjölds väg 14B, 752 37 Uppsala, Sweden; Child Health and Parenting (CHAP), Department of Public Health and Caring Sciences, Uppsala University, Husargatan 3, 752 37 Uppsala, Sweden; Department of Public Health and Caring Sciences, Uppsala University, Husargatan 3, 752 37 Uppsala, Sweden; Aging Research Center, Karolinska Institutet and Stockholm University, Tomtebodavägen 18 A, 171 65 Stockholm, Sweden; Research and Learning for Sustainable Development and Global Health (SWEDESD), Department of Women’s and Children’s Health, Uppsala University, Hammarskjölds väg 14B, 752 37 Uppsala, Sweden; Department of Women’s and Children’s Health, Karolinska Institutet, Tomtebodavägen 18A, 171 77 Stockholm, Sweden; Department of Global Public Health, Karolinska Institutet, Tomtebodavägen 18 A, 171 65 Stockholm, Sweden; Child Health and Parenting (CHAP), Department of Public Health and Caring Sciences, Uppsala University, Husargatan 3, 752 37 Uppsala, Sweden

**Keywords:** evidence-based health promotion, health education, health information, health literacy, intervention, epidemiology

## Abstract

Structural and contextual factors such as limited work and housing opportunities negatively affect the health and well-being of newly settled refugee migrants in receiving high-income countries. Health promotion initiatives aiming at strengthening health and integration have been tried out within the Swedish Introduction program for refugee migrants. However, longitudinal evaluations of these interventions are rare. The aim of the current study was to compare the effectiveness of a regular and an extended civic orientation course with added health communication and examine whether the latter would improve self-rated health and psychological well-being, health literacy and social capital among newly settled refugee migrants in Sweden. Pre- and post-assessment questionnaires were collected from the intervention group receiving the extended course (*n* = 143) and a control group receiving the regular course (*n* = 173). Linear mixed models and chi-square analyses showed a significant increase with a small effect size (0.21) in health literacy in the intervention group. However, there were no significant changes in emotional and practical support, general self-rated health or psychological well-being. The findings indicate that added health communication provided embedded in the civic orientation course can increase health literacy. However, further longitudinal studies are needed to confirm the sustainability of the observed effect and examine whether these short-term improvements in health literacy translate to long-term advances in health and integration.

**Contribution to health promotion**

- This study compared the effectiveness of a regular and an extended civic orientation course with added health communication for newly settled refugee migrants in Sweden.
- Linear mixed models and chi-square analyses showed a significant increase with a small effect size (0.21) in health literacy in the intervention group. No significant changes were observed in emotional and practical support, general self-rated health or psychological well-being.
- The findings indicate that added health communication provided within the civic orientation course for refugee migrants can increase health literacy. However, further longitudinal studies are needed to confirm the sustainability of the observed effect over time.

## BACKGROUND

In the past decade, countries in the European Union have seen an increase in the number of refugee migrants from war and conflict-torn countries such as Syria, Iraq, Eritrea and Afghanistan (WHO, 2018b). Even with declining numbers of migrants due to restrictive immigration policies applied in some countries, and the effect of the Covid-19 pandemic, the health of newly settled refugee migrants constitutes a major public health issue in European countries. In ecological terms, the health of migrants is determined by pre-peri and post-migration health status and life conditions (Zimmerman *et al*., 2011; Miller and Rasmussen, 2017). Refugee migrants are heterogeneous in terms of biology and hereditary factors, educational background and other pre-migration conditions and experiences, although they report similar migratory experiences and reasons for seeking refuge (Ben Farhat *et al*., 2018; Mangrio *et al*., 2018). Nevertheless, as a group, they often have poorer self-rated general health and mental health compared to the general population (Bas-Sarmiento *et al*., 2017; Alkaid Albqoor *et al*., 2020). Moreover, inequities between migrants and majority populations in, e.g., mental health outcomes persist over time in the new countries of residence (Blackmore *et al*., 2020; Honkaniemi *et al*., 2020), which highlights the importance of health promotion in the early post-migration phase, i.e. during the first 5 years in the country of resettlement.

In the post-migration phase, refugee migrants face structural and context-bound barriers that impact their health and well-being negatively; limited housing and work opportunities, restrictive integration policies, discrimination, isolation and acculturation stress are that examples of known factors adversely affect health in the resettlement phase (Porter and Haslam, 2005; Kastrup, 2016; Li *et al*., 2016; Juárez *et al*., 2019; Choy *et al*., 2021). In addition, migration may negatively affect individuals’ social capital, i.e. sharing, trusting and aiding relationships and networks (Kawachi I. SS, 2008), which are often disrupted by migration and take time to re-establish (Hassan *et al*., 2016; Sundvall *et al*., 2021). Similarly, a change of context and setting may lead to lower health literacy, defined as ‘*knowledge*, *motivation and competences to access, understand, appraise and apply health information in order to make judgments and take decisions in everyday life concerning healthcare, disease prevention and health promotion to maintain or improve quality of life*’ (Sorensen *et al*., 2012). Navigating a different health care system and language than one is used to may influence a person’s health literacy capacities, which is reflected in lower health literacy in newly settled migrant populations (Wångdahl *et al*., 2014, 2018). Social support and health literacy are important resources linked to better health outcomes and empowerment (Kawachi *et al*., 2008; Nutbeam, 2008) that in turn can buffer against post-migration stressors. Empirical studies on migrant populations have found that social capital could modify the effects of psychological ill health (Lecerof *et al*., 2016; Johnson *et al*., 2017), and low health literacy is associated with poorer access to health and health (Wångdahl *et al*., 2018; Ward *et al*., 2018).

The need to promote refugee migrants’ health outside of healthcare services is increasingly being recognized as important by local authorities, and stakeholders meeting refugee migrants in different capacities (Carlzén *et al*., 2016; Svanholm *et al*., 2020, 2022). Health promotion is understood as processes enabling people, families and communities to gain greater control over their lives, as defined by WHO Ottawa Charter for Health Promotion (1986). A salutogenic approach, promoting individual health resources and skills, is seen as a way to enhance resilience against post-migration stressors and increase the likelihood of successful resettlement and work integration (Carlzén *et al*., 2016; Svanholm *et al*., 2022). Good mental health is for instance associated with better learning outcomes (Asfar *et al*., 2019) such as language learning, which increases the likelihood of finding adequate employment, which in turn is a strong determinant for health and well-being (Drake and Wallach, 2020). This is echoed by WHO recommendations for health promotion activities designed for refugee migrants. The WHO stresses the importance of social determinants of health and recommends a multi-sectoral policy approach to health promotion rather than one focusing on the healthcare sector alone (WHO, 2018a).

Studies in the European region about health promotion programs both inside and particularly outside of the health care services are scarce, and there is a need for a more systematic evaluation and outcome assessment within the field (Diaz *et al*., 2017; WHO, 2018a). Moreover, few studies focus on refugee migrants in the early post-migration phase. Earlier global and European studies on health promotion initiatives outside of the health care system targeting newly settled refugee migrants include culturally or group-adapted educational health promotion (Ekblad and Persson-Valenzuela, 2014; Svensson *et al*., 2016; Pelters *et al*., 2022), effects of educational health promotion interventions (Eriksson‐Sjöö *et al*., 2012; Lecerof *et al*., 2017) and community-centered or community-led health promotion among specific refugee migrant groups (Hartwig and Mason, 2016; Salt *et al*., 2017; Asdigian *et al*., 2021).

### Health promotion within the Swedish Civic Orientation

Newly settled refugee migrants with a temporary or permanent residence permit take part in Civic Orientation (CO), an orientation course about Swedish society and living in Sweden. The CO is part of a 2-year mandatory Introduction Program that aims to facilitate work and integration of refugee migrants. Other components of the program are Swedish language training, vocational training and job-seeking counseling. Fulfillment of the program activities entitles a monthly welfare benefit.

The CO course is generally provided in the most common languages of participants and delivered by native-speaking teachers, referred to as civic communicators. Since 2020, the CO includes a minimum of 100 hours of dialogue-based teaching of learning modules spread over four knowledge areas; practical information about everyday life in Sweden, human rights and Swedish democratic values, how society is organized, and rights and obligations of the individual (Supplement 1). Health is included as a separate module focusing on how to care for one’s health and information about how the Swedish healthcare system works. Despite the lack of a clear mandate and support from national Swedish policymakers, initiatives have been launched on the regional and municipal level to promote the health of newly settled migrants. These initiatives, e.g. extended health communication embedded within the CO course, have been evaluated from the perspectives of participants and civic communicators (Svensson *et al*., 2016; Al-Adhami *et al*., 2021; Svanholm *et al*., 2022) and policymakers, and stakeholders (Carlzén *et al*., 2016; Svanholm *et al*., 2020, 2021). However, to our knowledge, only one prior study within the setting of CO has used a longitudinal design to investigate health outcomes and self-rated health (SRH) knowledge among newly settled migrants (Lecerof *et al*., 2017).

### Aim

The aim of the study was to compare the effectiveness of a regular and an extended CO course with added health communication among newly settled refugee migrants in Sweden and examine whether the extended CO course would improve SRH and psychological well-being, health literacy and social capital as measured by emotional and practical social support.

## METHODS

### Study design

The study had a quasi-experimental design with one control and one intervention group. The control group received the regular CO, and the intervention group the extended CO. The groups were assessed pre and post with the same measurements.

### Procedure and intervention delivery

At the time of the study, the regular CO in Uppsala County included 60 hours with a standard content of eight learning modules. The extended CO course consisted of 80 hours: the standard 60 hours plus 20 hours of added health themes (Supplementary Material 1).

Both the regular one and the extended version were delivered in the form of lectures, which were based on dialog, discussion and reflection (rather than one-way communication). This form was chosen to encourage participants to engage more, ask more questions and share their experiences. The course material was also designed to enable such an approach. For instance, images and film clips were used in the presentations and each session included some exercises (e.g. where to find specific information online). In addition, the course included site visits (e.g. visiting NGOs), and representatives from different NGOs and other stakeholders visited the class. The health and added health themes were spread throughout the course in both the intervention and control groups.

The civics communicators were fluent in Swedish and Arabic and had knowledge about Swedish society. They also had pedagogical training and other relevant experiences and knowledge about related course topics. A health educator was employed to train the civics communicators prior to the intervention. All civics communicators received 80-hour education including lectures and exercises by the health educator and external educators on topics such as food, exercise and lifestyle changes, sexual health and reproductive rights, mental health, gender equity, human rights in practice and violence in close relationships.

The study was carried out between January 2018 and September 2019 in six of eight municipalities within Uppsala County. The regular CO (i.e. control group) was rolled out first, followed by the extended CO (i.e. intervention group). The locations for data collection were selected in cooperation with the Office for Civic Orientation in Uppsala Municipality. The regular course was delivered over 8 weeks, and the extended one over 12 weeks. The study questionnaires were distributed during the first and last sessions of each CO course.

The questionnaire was a paper and pencil form. The post-assessment questionnaire included some additional questions about the course, which were not analyzed. All measures (see below) were available in Arabic as they had previously been translated from Swedish to Arabic by the research group and used in similar studies (Wångdahl *et al*., 2018; Al-Adhami *et al*., 2022). The original translations were done following scientific translations’ guidelines (Guillemin *et al*., 1993) and included back-translation, peer (expert-check) and pilot-testing with participants.

### Participants

The study participants consisted of Arabic-speaking newly settled adult refugee migrants with a residence permit attending CO courses. We used convenience sampling based on the availability of classes in Arabic and Arabic-speaking staff to administer the questionnaire. The inclusion criteria were enrollment in the CO course and speaking Arabic. The course participants were informed about the study both orally and in writing in their native language. Those who consented to participate were given the questionnaire that was filled out in the classroom.

**Fig. 1:**
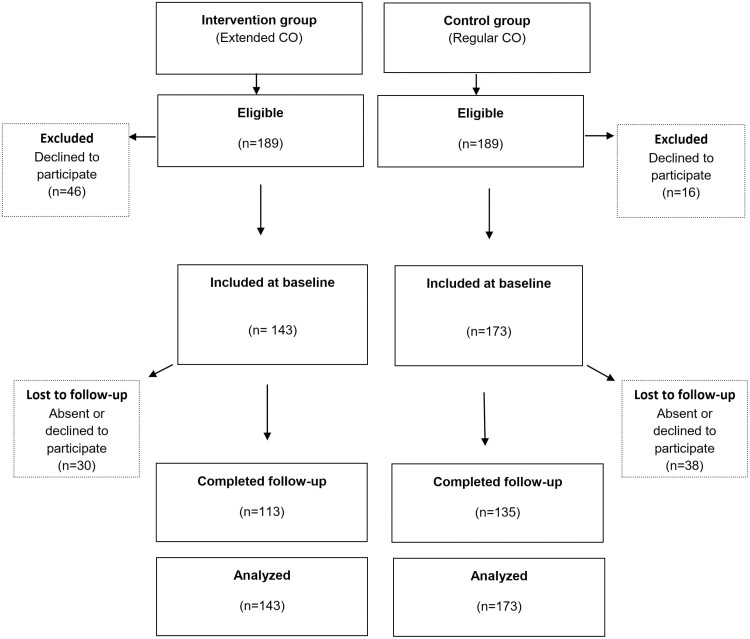
Flow chart for participation in the study

We visited 21 classes at baseline and follow-up. Arabic-speaking staff remained in the classrooms to assist those who needed support with reading the questions. Of 336 individuals eligible at baseline, 316 (94%) consented to participate in the study and completed the first questionnaire. Of those, 248 (74%) were present at the last session of the course and completed the post-assessment. Figure 1 shows the participation flow in the study.

### Measurements

The background variables consisted of sociodemographic questions about gender, age, educational level, country of birth, type of residence permit (temporary or permanent) and long-term illness. The latter was measured by the question ‘Do you have any chronic disease, or problems due to an accident or functional disability or other long-term health problem?’ with response alternatives no and yes.

We included the following outcome measurements in the study; SRH, psychological well-being, health literacy and emotional and practical social support (bonding social capital). The selection of the measurements was guided by social determinants of health as a framework and a salutogenic approach to health promotion.

### Self-rated health

*SRH* was measured using the question ‘How do you assess your overall health status?’ which measures general physical and emotional health. The item has been widely used as an indicator of a person’s subjective general perception of their health (Nielsen and Krasnik, 2010). It has five response alternatives: very good, good, neither good nor bad, bad and very bad. In the current study, we dichotomized the responses into good (the first two alternatives) and poor/less than good health (the last three alternatives) (Chandola and Jenkinson, 2000; Johansson *et al*., 2015).

### Psychological well-being

*Psychological well-being* was measured using the 12-item version of the General Health Questionnaire (GHQ-12). The GHQ-12 has been used in different countries and settings to assess non-psychotic mental health problems such as anxiety, depression and loss of confidence over the past few weeks (Goldberg *et al*., 1997). Each item has a four-point scale ranging from 0 (e.g. better than usual) to 3 (e.g. much less than usual), generating a sum score of 0–36. Higher scores indicate poorer condition. The GHQ-12 has been found to have satisfactory validity and cross-cultural sensitivity (Goldberg *et al*., 1997; Lundin *et al*., 2017). In the current study, the scale had a Cronbach alpha of 0.83.

### Health literacy

*Health literacy* (HL) was assessed using the European Health Literacy Survey Questionnaire 16 (HLS-EU-16) (Pelikan and Ganahl, 2017b), which is the shorter 16-item version of the original Health Literacy Scale-EU-47. Each item is rated on a four-point scale (very easy, easy, difficult and very difficult). Following the guideline of the scale (Pelikan and Ganahl, 2017a), the ‘difficult’ options are assigned a value of 0 and the ‘easy’ options a value of 1, giving a possible range of 0–16 for the sum score. Higher scores indicate better HL, with scores 13–16 signifying likely sufficient HL.

The HLS-EU-16 has a high correlation with the original version (*r* = 0.82) (Pelikan *et al.*, 2014) and has been found to have adequate psychometric qualities including internal consistency, reliability and construct validity in the general and migrant populations in Europe (Pelikan and Ganahl, 2017b; Mekhail *et al*., 2022). In the current study, the scale had a Cronbach alpha of 0.84.

### Social support

Social support was measured with a number of single-item questions derived from the theoretical framework of social capital (Kawachi I. SS, 2008). The questions have been theoretically and empirically validated in different settings (Svensson *et al*., 2013; Johnson *et al*., 2017). Bonding social capital (trust and sharing between individuals within a homogenous group), i.e. social support, was measured by emotional and practical social support questions.

*The emotional social support* was phrased as ‘Do you have anybody whom you can share your deepest feelings with and confide in?’ with yes and no response alternatives. *Practical social support* was phrased as ‘How many people in your surroundings can you easily ask for help with everyday tasks’ and dichotomized into none and one or more persons.

### Power analyses

Retrospective power analysis indicated that the sample size of 316 participants in this study enabled us to detect a small effect size of 0.16 (Cohen’s *d*) with alpha set at *P* < 0.05 and power at 0.80 (Faul *et al*., 2007).

### Statistical analysis

The percentage of incomplete items varied between 0 and 31 (missing at the individual item level). Of 316 records, 222 (70%) were incomplete (had missing on at least one of the 73 variables). Missingness was related to lower education and reporting low emotional support. We used the default settings in SPSS 28 to impute the missing values at the item level under conditional specification. Using multiple imputations, we created and analyzed 100 multiply imputed datasets. We report pooled results unless specified otherwise.

We used descriptive statistics to describe the sample, and a series of chi-square tests and ANOVAs to compare (i) the intervention and control groups at baseline and (ii) the baseline characteristics of those who stayed in the study and those who dropped out (those with and without data at post-assessment).

To investigate differences over time (baseline to follow-up) between the two groups, we used a linear mixed model for the scales (HLS and GHQ-12; including age as a covariate because there was a significant age difference between the intervention and control groups at baseline) and chi-square test for the categorical variables (self-rated general health, emotional and practical social support). *F* and *P* values for categorical values were calculated based on the D2 statistic for pooling chi-square values. We used a one-sided *P*-value for the outcome analysis because we expected the intervention (the extended CO) to be superior to the control (regular CO).

All continuous outcome data were examined through visual inspection of histograms and were found to be approximately normally distributed. We conducted the analyses using SPSS 28 and R 3.5.2.

### Ethical consideration

Initially, the participants received both written and oral information about the study in their native language, Arabic. The information included the aim of the study, examples of questions in the survey, data handling and storing procedures and regulations, and how the information would be disseminated. The participants were then informed that they could discontinue their participation at any time during the study, without any questions or consequences. Second, written informed consent was collected from all participants before handing out the questionnaire. The study was approved by the Swedish Ethical Review Board in Uppsala (registration number 2017/437).

## RESULTS

### Sample characteristics at baseline

Participants’ characteristics at baseline are presented in Table 1. The original and pooled data were almost identical. The sample consisted of more women than men (63% and 55% in the intervention and control groups, respectively). Participants ranged in age from 20 to 65 years with participants in the intervention group (*M* = 35.0, SD = 9.6) being slightly younger than the control group (*M* = 38.3, SD = 11.7) (*P* < 0.05). The educational level was distributed relatively evenly across the four categories (0–6, 7–9, 10–12 and more than 12 years of education). The high majority of participants were born in Syria (over 78%) and had a permanent residence permit (over 62%). The average time spent in Sweden was 3 years (not shown in the table). Most participants had no long-term illness (72% and 68% in the intervention and control groups, respectively). There were no statistically significant differences between the intervention and control groups on any of the background or outcome variables at baseline except age.

**Table 1:** Characteristics of intervention and control group at baseline for original and pooled data

	Original data								Pooled data					
	Intervention			Control			Group comparison		Intervention		Control		Group comparison^a^	
	Valid *n* (missing)	*n*	%	Valid *n* (missing)	*n*	%	χ^2^/*F*	*P*	*n*	%	*n*	%	*F*	*P*
Gender	143 (0)			173 (0)										
Man		53	37.1		78	45.1	2.08	0.169	53	37.1	78	45.1	*F*_(1,Inf)_ = 1.76	0.185
Woman		90	62.9		95	54.9			90	62.9	95	54.9		
Age (range 20-65)	141 (2)	M 35.1	(SD) (9.6)	172 (1)	*M* 38.3	(SD) (11.7)	*F*_(1, 311)_ = 6.74	0.010	M 35.0	(SD) (9.6)	M 38.3	(SD) (11.7)	*F*_(1,Inf)_ = 6.98	0.008
Country of birth	135 (8)			162 (11)										
Syria		106	78.5		133	82.1	0.60	0.465	111.5	78.0	140.7	81.3	*F*_(1,Inf)_ = 0.35	0.557
Other		29	21.5		29	17.9			31.5	22.0	32.3	18.7		
Education	142 (1)			170 (3)										
0–6		30	21.0		27	15.9	3.02	0.388	30.4	21.2	28.0	16.2	*F*_(3,Inf)_ = 0.94	0.418
7–9		37	26.1		37	21.8			37.3	26.1	37.9	21.9		
10–12		38	26.8		52	30.5			38.3	26.8	52.8	30.5		
More than 12		37	26.1		54	31.8			37.1	25.9	54.4	31.4		
Type residence permit	133 (10)			159 (14)										
Permanent		82	61.7		107	67.3	1.01	0.328	88	61.5	116.6	67.4	*F*_(1,Inf)_ = 0.86	0.354
Temporary		51	38.3		52	32.7			55	38.5	56.4	32.6		
Long-term illness	140 (3)			168 (5)										
No		101	72.1		114	67.9	0.67	0.456	102.9	72.0	116.7	67.5	*F*_(1,Inf)_ = 0.54	0.462
Yes		39	27.9		54	32.1			40.1	28.0	56.3	32.5		
General health (SRH)	142 (1)			172 (1)										
Good		93	65.5		109	63.4	0.15	0.724	93.1	65.1	109.8	63.5	*F*_(1,Inf)_ = 0.03	0.854
Poor		49	43.5		63	36.6			49.9	34.9	63.2	36.5		
Psychological well-being	137 (6)	M 9.39	(SD) (5.96)	160 (13)	M 9.66	(SD) (6.18)	*F*_(1, 295)_ = 0.15	0.697	M 9.46	(SD) (6.01)	M 9.76	(SD) (6.33)	*F*_(1,Inf)_ = 0.55	0.459
Health literacy	130 (13)	M 10.57	(SD) (3.64)	156 (17)	M 10.46	(SD) (3.82)	*F*_(1, 284)_ = 0.07	0.797	M 10.57	(SD) (3.62)	M 10.45	(SD) (3.84)	*F*_(1,Inf)_ = 0.08	0.772
Emotional support	138 (5)			168 (5)										
High		113	81.9		132	78.6	0.52	0.565	116.6	81.5	135.3	78.2	*F*_(1,Inf)_ = 0.32	0.573
Low		25	18.1		36	21.4			26.5	18.5	37.7	21.8		
Practical support	140 (3)			167 (6)										
High		126	90.0		154	92.2	0.47	0.547	128.8	90.1	159.4	92.1	*F*_(1,Inf)_ = 0.20	0.654
Low		14	10.0		13	8.8			14.2	9.9	13.6	7.9		

^a^For categorical variables, *F* and *P* values are based on the D2 statistic for pooling chi-square values. Degrees of freedom for the denominator are approximated as infinite.

### Attrition at post-assessment

The internal dropout rate, i.e. the rate of dropouts between baseline and follow-up, was 21% in the intervention group and 22% in the control group. We conducted sensitivity tests (Pearson chi-square and ANOVA) to investigate potential differences between those who dropped out and those who had remained in the study. We included all background and outcome variables and found only two significant differences between those who remained in the study and those who dropped out. In the intervention group, younger participants and those who had low practical support were more likely to drop out (*F *= 4.28, *P *= 0.040 and *F *= 1.71, *P *= 0.031, respectively).

### Intervention outcome

Interaction effects between the groups (intervention and control groups) and time (pre- and post-assessment) are shown in Tables 2 and 3. Significant interaction effects between group and time were observed for HL (*F* = 0.21, *P* = 0.032, Cohen’s *d* = 0.21). Investigation of the means showed that from pre- to post-assessment, participants in the intervention group reported more improvement in HL compared to the control group. The pattern of change over time in the two groups was not significantly different for any of the other outcomes. These findings indicated that the added health communication module resulted in small improvements in HL post-intervention, but it did not contribute to improved emotional and practical support, general SRH or psychological well-being.

**Table 2:** Intervention effects; pre- to post-assessment differences for continuous variables (pooled data) with age included as covariate

	Intervention group (*n* = 143)		Control group (*n* = 173)		Main effect of time		Main effect of group		Interaction group × time		
	Baseline	Follow-up	Baseline	Follow-up							
Outcomes	*M* (SD)	*M* (SD)	*M* (SD)	*M* (SD)	*F*	*P*	*F*	*P*	*F*	*P*	Cohen’s *d*
Psychological well-being	9.46 (5.99)	7.33 (4.93)	9.76 (6.31)	7.35 (4.99)	1.13	<0.001	0.13	0.635	0.07	0.338	0.05
Health literacy	10.57 (3.66)	12.80 (2.90)	10.45 (3.83)	11.86 (3.46)	-0.91	<0.001	−0.27	0.118	0.21	0.032	0.21

**Table 3:** Intervention effects; pre- to post-assessment differences for categorical variables (pooled data)

	Intervention group (*n* = 143)						Control group (*n* = 173)								
	Negative change		No change		Positive change		Negative change		No change		Positive change				
Outcome	*n*	%	*n*	%	*n*	%	*n*	%	*n*	%	*n*	%	*F* ^a^	df	*P*
General health (SRH)	13.8	10	100.3	70	28.9	20	13	8	133.4	77	26.6	15	0.89	2, 2966	0.411
Emotional support	18	13	111.8	78	13.2	9	14.6	8	135.5	78	22.9	13	0.99	2, 2776	0.374
Practical support	7.1	5	124.9	87	10.4	7	7.2	4	161.4	93	4.4	3	2.15	2, 10995	0.116

^a^ *F* is the pooled chi-square statistics.

Significant effects of time alone were observed for psychological well-being and HL (*F* = 1.13, *P* < 0.001 and *F* = −0.91, *P* < 0.001, respectively). This shows that following the CO course, participants in both conditions reported an improvement in their psychological well-being (reduction of psychological distress) as well as HL.

For the categorical outcome variables (i.e. SRH and social support measures of emotional and practical support), the vast majority of participants reported no change over time (70–93%), while few reported positive or negative changes (3–20% and 4–13%, respectively).

## DISCUSSION

The study aimed to examine whether an extended CO course with added health communication improved self-rated and psychological well-being, HL and social capital as measured by emotional and practical social support. The results showed that the extended course led to greater improvement in HL. No significant differences were found for participants in the regular versus extended course in other outcomes i.e. SRH, psychological well-being and emotional and practical support.

We assumed that the added health communication module would likely lead to increased HL and social capital at post-intervention, but that general and psychological health might take longer to be improved. This assumption was based on theoretical understandings of educational health promotion interventions; increased knowledge could lead to sustainable change in behavior if factors such as motivation, self-regulation and resources are present (Kwasnicka *et al*., 2016), but for the change to have an effect on primary health outcomes requires time. Our assumption was also in line with results from a previous longitudinal study on a health promotion program conducted within the realm of the CO (Lecerof *et al*., 2017), showing an increase in health knowledge following the program but not in general health and psychological well-being. It is theorized that educational health promotion actions may lead to improved overall health outcomes through two pathways: (i) increased HL skills and motivation and (ii) intermediate health outcomes such as improved health-seeking behaviors and lifestyle changes (Nutbeam, 2000). A prior qualitative study on a health intervention within the CO showed that participants had an appreciation for the health content and could express what they learnt, and in which ways they were inspired to change, i.e. had increased their knowledge and motivation linked to HL (Al-Adhami *et al*., 2021). Our findings on improved HL skills are also in line with other studies on group-adapted educational health promotion initiatives (Ekblad and Persson-Valenzuela, 2014; Fernández‐Gutiérrez *et al*., 2018). However, even if there were a significant increase in HL, a follow-up study would be needed to confirm robust longstanding effects. Furthermore, as the effect size (Cohen *d* = 0.21) is considered small (Cohen, 2013; Lakens, 2013), it needs to be weighed against added costs and other priorities that the newly settled might have within the scope of their introduction activities. Nevertheless, the results regarding HL show that the mean of the intervention group was approaching the level for sufficient HL (12.80 with the threshold being 13). Furthermore, the mid-term gains include improved healthcare utilization, health behaviors, disease prevention and empowerment (Nutbeam, 2000, 2008; Sorensen *et al*., 2012) vital for the resettlement process. The findings also confirm that HL interventions within the CO have the potential of enhancing participants’ knowledge and skills. The latter is in line with WHO policy recommendations regarding health promotion for refugees and migrants that mention investing in language support and HL initiatives to develop personal skills (WHO, 2018a).

Regarding social capital, a person’s active engagement in interpersonal relationships, as in a group activity or course, has been linked to better health outcomes by way of increased received or perceived social support (Cohen, 2004; Kawachi *et al*., 2008). In a previous qualitative study within the setting of CO, participants reported having gained new social contacts (Al-Adhami *et al*., 2021), which can be hypothesized as having increased their social support network. Based on this, we expected a moderate positive change in social support in the current study, due to a prolonged interaction with others in the intervention group. However, we did not observe an effect on either emotional or practical social support. At baseline, the proportion of those reporting high emotional and practical support was around 80% and 90% for the respective measures. This might have led to a ceiling effect (Garin, 2014), i.e. there was not much room for improvement. There are other possible reasons, one being the use of single-item social capital measures that might not have been sufficient to detect change. Another possibility is that the intervention was limited in scope, i.e. the prolonged interaction with others was too short to show any significant difference compared to the regular course.

### Methodological considerations

The high response rate at baseline (94%) could imply that participants felt somewhat obliged to participate despite being informed about the possibility to opt out without any consequences. However, there was no advantage to participating and no apparent ‘right answer’ with a survey including a variety of items and questions. The availability of the questionnaire in Arabic and the self-administered method minimized the occurrence of social desirability bias, as no further translation of the questions was necessary. In a similar study, the use of translators and face-to-face interaction was hypothesized to create such bias (Mekhail *et al*., 2022). In general, we believe that the translated questionnaire, and its delivery within the CO (a mandatory introduction activity), was a strength as it maximized the opportunity of participation for a group that is considered ‘hard to reach’ and therefore often underrepresented in health promotion programs and research studies (Shaghaghi *et al*., 2011).

A general limitation is the quasi-experimental design of the study. An RCT would have provided more robust conclusions. However, an RCT was not feasible due to logistical and financial limitations in the project.

The outcome measurements in the study are self-reported. This carries certain limitations such as the influence of subjective traits on ways to respond (Paulhus and Vazire, 2007) and that questions might be understood differently depending on for instance educational attainment. This, however, has to be weighed against the practicality of using them and the fact that they are established measurements, tested for validity and reliability and used across different settings. Another limitation was that the single-item measures of social capital might not have been sensitive enough to detect intervention effects.

As for the HLS-16 scale, a recent study reported that foreign-born respondents found a few of the questions to be advanced and somewhat unclear (Mekhail *et al*., 2022) (which was not reported by the native-born). This difficulty could also apply to the current study. However, the possible effect of this on the outcome of our study should be less of a concern as all the respondents were foreign born, compared to studies that include native- and foreign-born respondents.

## CONCLUSION

Educational health promotion interventions targeting newly settled migrants in the early post-migration phase could be beneficial as they may increase HL, which in turn is linked to improved health-seeking behavior, better health outcomes and empowerment. However, longitudinal studies with multiple follow-ups are needed to confirm the sustainability of change in HL as well as potential long-term health outcomes.

## Supplementary Material

daae015_suppl_Supplementary_File_1
