# Supplementary figures and images for: Can extended health communication improve newly settled refugees’ health literacy? A quasi-experimental study from Sweden
Source: Health Promot Int. 2024 Mar 2;39(2):daae015. doi: 10.1093/heapro/daae015 (PMC10908352; doi:10.1093/heapro/daae015)

**Supplementary file 1. Content of regular and extended CO course**


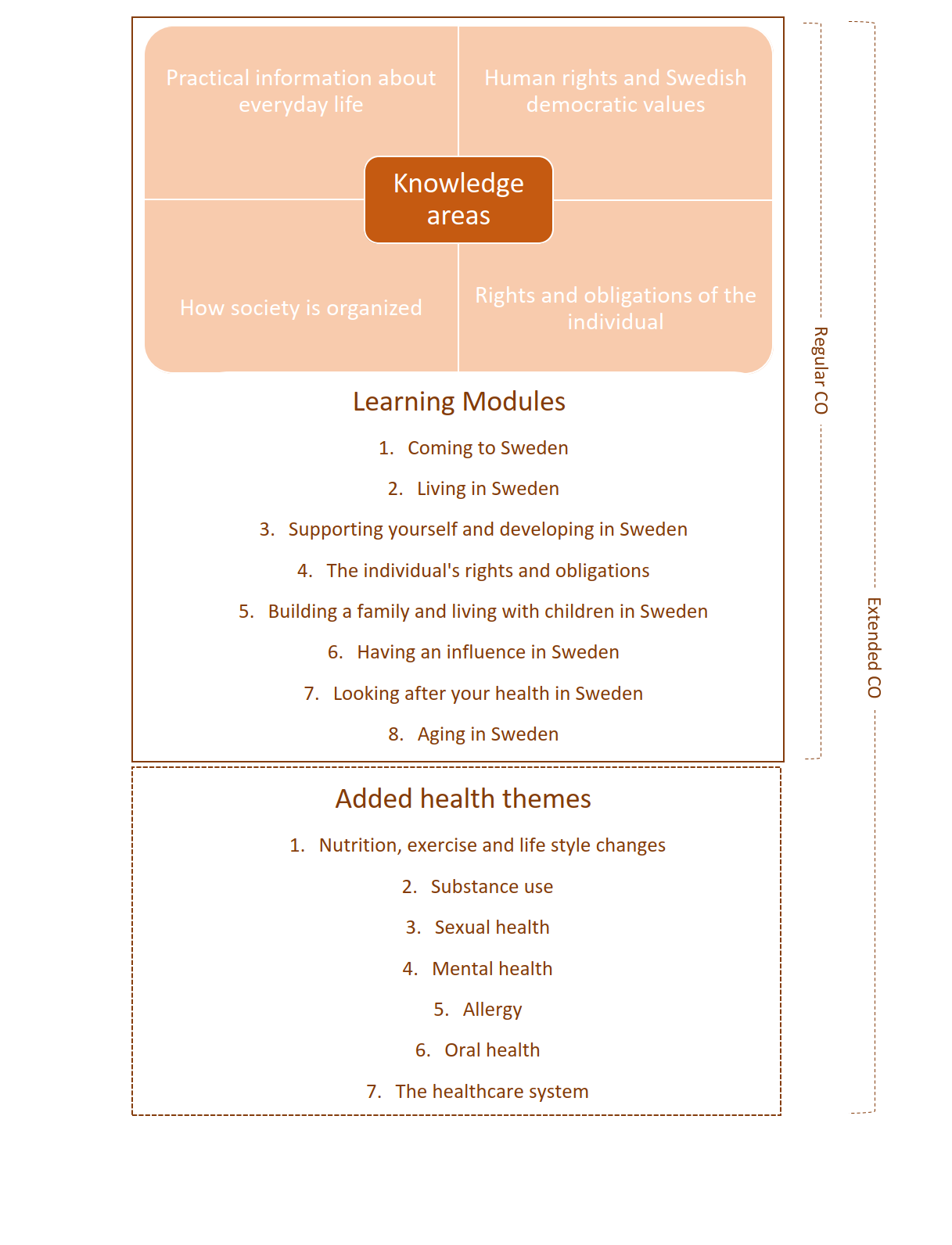

Supplement: daae015_suppl_Supplementary_File_1 [file daae015_suppl_supplementary_file_1.docx]
